# Multilayer perceptrons, Hopfield’s associative memories, and restricted Boltzmann machines

**DOI:** 10.1186/1471-2202-15-S1-P223

**Published:** 2014-07-21

**Authors:** Shin-ichi Asakawa

**Affiliations:** 1 Center for Information Sciences, Tokyo Woman’s Christian University, 2-6-1 Zempukuji, Suginami-ku, Tokyo 167-8585, Japan

**Keywords:** multilayer perceptrons, Hopfield’s associative memories, restricted Boltzmann machines, one algorithm hypothesis

## 

This study was intended to describe multilayer perceptrons (MLP), Hopfield’s associative memories (HAM), and restricted Boltzmann machines (RBM) from a unified point of view. Despite of mutual relation between three models, for example, RBMs have been utilizing to construct deeper architectures than shallower MLPs. The energy function in HAM is analogous to the Ising model in statistical mechanics, and it connects microscopic physics to thermodynamics. The canonical partition function *Z* in the Boltzmann distribution is also utilized RBMs. Asynchronous updating and contrastive divergence (CD) based upon Gibbs sampling is also related. Therefore, it seems to be worth considering these three models within a common framework. This attempt might lead to “one algorithm hypothesis.”, which insists that our brains might rule a single but universal rule.

An algorithm, which someone could find out in a region, may be applicable to other regions. Multilayer perceptrons (henceforth, MLP) are feed forward models for pattern recognition and classification. Hopfield proposed another kind of neural network models for associative memory and optimization (HAM). Hiton adopted the restricted Boltzmann machines (RBM) in “Deep Learning” in order to construct deeper layered neural networks. The energy employed in RBMs are elicited the generalized EM algorithm, which was closely related to the energy employed by HAM. In spite of other various differences, see Table 1, it is worth considering to compare among them. At least, an attempt is worth attempting to explain all of them in a unified terminology.

**Table 1:** Summary of MLP, HAM, and RBM

	Architecture	Learning	Flow Direction	Within Layer Connection	Update
MLP	Layered	Gradient Descent	Feed Forward	No	Synchronous

HAM	Mutual	No (EM Algorithm)	No Directions	Yes	Asynchronous

RBM	Layered	Contrastive Divergence	Mutual	No	Synchronous

HAM and RBM have symmetrically weighted connections, *w_ij_* = *w_ji_*, although generalized Boltzmann machines can not satisfy this constraints. Similarly, there are no feedback connections in MLP in general. When we denote a connection weight from *j*-th unit to *i*-th unit as *w_ij_*, *w_ij_* ∈ R, *w_ji_* = 0 in MLP. When we consider a merged weight matrix W, all the models can be considered as identical.

The construction methods adopted by Deep Learning are based upon RBMs. One of key concepts to success for constructing multilayer deep architecture is the non–linearity, because units in hidden layer in RBMs are binary. The non–linearity seems to play an important role to construct deep architecture. When we suppose to abandon CD and binary feature, multilayer architecture might replace one weight matrix W = W_1_W_2_… W*p*. Also, we can consider a thought experiment with only one hidden unit in RBM. If *h* = 0, then there are no meanings at all. If *h* = 1, then it must be an identity mapping, or at least, it might be extract the eigenvector vector corresponded to the maximum eigenvalue value in data matrix X. This might be equivalent to the algorithm proposed by Oja (1985). Since Deep Learning architecture network models trained via RBMs have no within layer connections, we might not be able to reject a possibility that a hidden unit *hi* might be trained to detect exactly the same features as another hidden unit *hj*. In order to avoid these situations, we must prepare a large number of binary hidden units more than the entropy being involved in input data set. RBM has no assumptions about within layer connections, it might success to detect important features among data matrix via CD. However, this constraint might weaken slightly, when we would introduce the EM algorithm to be estimate the states of latent variables, and an online algorithm of HAM. This might bring us to an idea “semi restricted Boltzmann machines.”

